# Actinomycosis1Read before the twenty-first regular meeting of the Missouri Valley Veterinary Medical Association.

**Published:** 1899-12

**Authors:** J. B. Wright

**Affiliations:** St. Joseph, Mo.


### ACTINOMYCOSIS.[1]

By J. B. Wright, D.V.S.,
ST. JOSEPH, MO.

Actinomycosis is due to the entrance into the tissues of a vegetable parasite named the actinomyces. It is characterized by the presence of suppuration and inflammatory new formations in various tissues. It has been seen in the pig, ox, horse, sheep, and also in man, but it attacks bovines by preference. For a long time this disease has been described under the name of osteosarcoma, or tumors of the jaws, due, as was supposed, to action of various kinds, such as a kick or prod with the horn. Some authors described this disease as of a cancerous or tuberculous nature. Later these facts were dispelled by Perroncilo and Revolta, when they discovered in these tumors the presence of a parasite divided into short filaments or club-like in shape. About two years later Bollinger began his first researches on these tumors on the jaws of the ox, and demonstrated a cauliflower-like parasite which the botanist Hartz, after a great deal of study, named the actinomyces. From thence we have the name actinomycosis. Since then numerous observations have been made, and we find Nocard recognized this disease for the first time in 1884. We are told that the same alterations are met with in other tissues of the body.

Since Nocard's investigation numerous observations have been made in the study of the actinomyces on the various animals affected. Actinomyces belongs to the vegetable kingdom, and is sometimes known as the ray fungus. It is common on all grasses, but seems to have a preference for the barley-straw. On microscopical examination we find it presents itself in yellowish masses, club-like in shape, and seems to form in groups, resembling almost a perfect rosette, the spindles or tail interlocking with one another. In certain tumors these enlargements are absent, the actinomyces being represented by a mass of ramified mycelium. These fungi often undergo calcification. The actinomyces

[1] Read before the twenty-first regular meeting of the Missouri Valley Veterinary Medical Association.

is colored by the methods of Gram and of Weigart. The cultures are obtained in the presence and in the absence of air. Actinomyces can be developed in different mediums—glycerin, serum, and also on potato culture. They develop in rounded colonies, in pale white masses, which afterward become yellowish or reddish. Inoculation transmits with difficulty the infection to the calf, rabbit, and guinea-pig. (Temperature favorable for the development of the culture is between 32° and 37° C. Cultures killed in ten minutes at zero.)

How does infection take place? The actinomyces may invade the animal economy in various ways, but principally through inoculation coming in contact with wounds in the mouth, diseased teeth, etc. Infection may also take place through the respiratory tract and through wounds of the skin. Generalized actinomycosis may take place through the circulation. Adult bovines are the animals particularly affected, although the disease attacks the horse, pig, sheep, and man. The affection is very rare in the horse and sheep. We also find it in the deer.

The disease has been for a long time known in Europe under different names for the regions affected, but with the same clinical study. For instance, in England we find it under the name of "woody tongue" where the tongue is affected, and lumpy jaw where the head and neck are affected. We also find in Europe and North America an epizoötic cancer of the tongue similar to actinomyces. The numerous works written on actinomycosis show that the disease can be reproduced on the Old and New Continent, with a very irregular development of the different localizations, such as the jaw, tongue, pharynx, etc.; but I must say a preponderance of statistics shows that the maxillary bones of the head of the ox are the places most principally affected and observed. From my own personal observations I am inclined to believe that localizations of the lungs and viscera are almost exceptional, although it has been currently reported by several authors. Quoting from Zuill, I notice that of 105 cases the maxillæ bones were affected in 51 per cent., the tongue in 29 per cent., and the larynx and trachea in 6 per cent. In France and Germany the maxillæ bones were the most observed. In Italy all the clinical forms are met with. In the county of Norfolk, England, 8 per cent. of the cattle affected the localizations were on the jaws and tongue (Cruikshank). In Russia the disease abounds in certain regions and in all centres. In this country actinomycosis abounds to a high degree. Hundreds of cattle are condemned yearly in the different markets. Three per cent. of the cattle shipped from Canada to England show the lesions.

From the above the question arises, How are we to study the different anatomical alterations?

Actinomycosis in Bovines. *The Jaw.* The localizations on the jaws correspond to tumors; this is the most commonly observed. The disease appears as an inflammatory swelling which first begins somewhere along the lower or upper jaw or on the side of the face. It is often accompanied by a sensibility of the region and difficulty in mastication. It grows slowly, and takes quite a long time before it reaches any size. Abscesses which open to the exterior emit a creamy, thick, doughy-like pus mixed with small, yellowish grains. Put a probe into the orifice and we will discern a regular fistulous tract, separated by soft tissues. (One peculiar aspect of this disease is the fact that the pus has no odor, and it will come away in strings.) The teeth become loose; in course of time mastication becomes slow and one-sided; these fistulous openings are surrounded by black granulations, often the size of a cocoanut; the animal becomes emaciated; grave functional troubles arise, and unless sacrificed will succumb.

*The Tongue.* The first symptoms consist of the difficulty of the animal in prehension. Mastication is slow and incomplete. The tongue cannot be extended from the mouth. It is tumefied and painful, and an abundant salivation. At this stage we find the tongue hard, rigid, and voluminous. Here we get the name woody tongue, and showing all over little knots of a yellowish color, from the size of a pea to a goose-egg, embedded in the mucous membrane. Here we observe the appetite is failing, the animal becomes emaciated, the disease is rapid, and at the expiration of a few weeks the animal succumbs to inanition if not sacrificed.

*The Pharynx.* The disease in this region is discovered by direct exploration of the mouth. These tumors, when found, are fibrous in consistence, and are from the size of a pea to a nut. These tumors generally entail a difficulty of deglutition and respiration, and are sometimes accompanied by a cough. We often find on exploration tumors of the adjacent glands. Owing to the difficulty of swallowing and painful respiration the animal emaciates rapidly and ought to be killed.

*The Neck.* Symptoms vary according to the seat of the regions involved. Sometimes we find the lesions are seated in the superior part of the shoulder or under the skin, the walls of the pharynx, œsophagus, glands, etc. In all cases there exists a diffuse tumefaction, which little by little becomes hard, painful knots, and finally discharge a thick, creamy, yellowish-looking pus, rich in actinomyces. If not relieved the cavity cicatrizes with great difficulty, the suppuration penetrates more deeply, fistulous tracts are formed, connecting different regions; the skin thickens to three times its natural size, surrounded by soft tissue, which suppurates and discharges pus rich in actinomycotic grains.

Outside of the lesions in the jaws, tongue, and pharynx we may find actinomycosis in any of the glands or other tissues of the body.

On the lips actinomycosis may be confounded with aphthous fever in calves. The tumors appear under the mucous membrane of the size of a pea to the size of a nut. They consist of a caseous, purulent centre, with fluctuating points, and on squeezing will be found to contain the actinomycotic grains.

In the respiratory passages we find the tumors infest the walls of the trachea, which cause trouble in respiration, varying, of course, to the size and seat of the tumors.

*The Lungs.* Although the localizations have been currently reported in Europe, I believe they are exceptional in this country, and from my own personal observation and from numerous inquiries I have seen only one case, and it presented one large tumor on the left lung, which, when cut open, emitted a characteristic pus of a yellowish color, sticky and of a doughy consistency, rich in actinomycotic grains. From this tumor little fistulous tracts were observed all through the lung substance, and at the end of each tract small tumors were found showing the same characteristics. The mediastinum glands were also found to be affected as well as some of the other glands of the body. A thorough examination of the skin and head of this carcass was made, but failed to find any exterior lesions. Sometimes the evolution resembles all the characteristics of tuberculosis. The symptoms are identical, and can be recognized only by the microscope.

Actinomycosis has been found in the mammary glands. Its evolutions also resemble tuberculosis.

Outside of its localizations on the maxillary bones it has been observed in the vertebræ and sternum. Here it invades the cancellated structure, and by reproducing and developing it causes the bony tissue to dilate and destroy, or the cancellated tissue may be absorbed. The growth is of a dense, fibrous consistence, and as the case progresses a spongy appearance of the bone appears, which shows centres of actinomycotic grains. Mystrom, in 1895, mentions a case of enzoötic actinomycosis developed in the pharyngeal region among young bovines maintained in the same pasture.

Generalized actinomycosis, although being very rare, is still possible. When the glands contiguous to the actinomycotic lesions become charged with the disease, then it is possible we may find a contamination of the whole system. As the glands become invaded with the actinomyces a spongy appearance is observed. In course of time the gland tissue is destroyed and the actinomyces become liberated in the circulation, and if a fruitful place of development is found characteristic lesions may be seen. Unless destroyed by nature's elements I am doubtful whether actinomycosis can be developed through the intestinal tract, as I believe the gastric juice of the stomach kills the germs or at least destroys their virulence or potency. My attention was called to a large umbilical tumor on a steer at one of the abattoirs. On the tumor being removed and dissected macroscopical examination showed all the characteristic lesions of actinomycosis. No other lesions were seen.

SWINE. This disease has been observed in the larynx, tongue, and mammary glands. These tumors are hard and painful, and they afterward unite in a large mass. Sometimes they discharge a creamy, thick pus, and on examination it will be found to contain actinomyces. They often become very large. Exceptional localizations have been found in the lungs. Knolle reports a case of generalized actinomycosis, the parasites being found in the tongue, serous membranes, liver, and vertebræ.

SHEEP. Actinomycosis in the sheep is rare.

HORSE. Actinomycosis has been found in the horse in nearly all centres, but it attacks, as in bovines, the bones by preference. In the tumors of the jaws we find a deliminated mass, creating an abscess, and persistent fistulous tracts, with a painful tumefaction of the parts involved.

In actinomycosis of the tongue the same alterations take place as in the ox. In actinomycosis of the neck the localizations present themselves as in the ox. Right here I would call your attention to the fact of the peculiar similarity between this disease and fistulous withers of the horse. Do we not find the same characteristics in the two diseases? I say, emphatically, we do; and permit me to suggest a thought that when we find persistent and troublesome fistulous withers, think of actinomycotic lesions, and medicate accordingly. In some cases of scirrhus cord actinomyces has been found.

Treatment. When this disease was first discovered treatment was thought to be of no avail, but in 1885, Rasmussen observed that in man the iodide of potash treatment clearly demonstrated a recovery. We are indebted to Nocard, for in 1892 he communicated his researches and demonstrated the marvellous effects of the iodide treatment on woody tongue.

In this country an official commission was charged to investigate the same subject. Out of a total of 185 animals affected on the jaws and parotid regions, 131, or 71 per cent., recovered; 53 animals affected in a less degree completely recovered after treatment. The treatment is administered by the digestive tract in doses of from two to four drachms daily. It should be continued for about twelve or fifteen days and then stopped for a short interval, when, if the lesions still persist, medication again may be commenced. Iodism is little to be feared. The lesions of the tongue are more easily cured than the osseous lesions. Of course, when the lesions withstand all internal remedies then surgical interference is necessary, combined with the internal treatment. Tincture of iodine externally is beneficial in some cases.

In the prophylaxis of actinomycosis we ought to isolate, as far as possible, diseased animals from the well ones until recovery is possible. We ought to cover the wounds with some kind of covering, to prevent the pus from falling on the grass or straw, and when cleansing wounds to burn or sterilize, as far as possible, all that has come in contact with the wound. Statistics do not show that this disease has been transmitted from animal to man, and although it is improbable, it is not impossible. On the other hand we have precise observations that the disease in man has been produced by vegetables, grasses, etc., and it follows, as in animals, the same procedure. Contagion from animals to man is then doubtful (unless through inoculation or vaccination), but we ought to use all necessary hygienic precautions so that no infectious matter may be carried. Washing the hands in water after being exposed to actino suppurations will be enough to eliminate all danger of infection. As contagion from animals to man is then doubtful, we ought to give the human family the benefit of the doubt, and reject for consumption all meat suffering from generalized actinomycosis or where we find a fistulous tract opening into the mouth, and where the glands contiguous to the wound are diseased.

*Discussion.*

Dr. Stewart: Owing to the absence of Dr. Bennett, who was announced to open the discussion of this paper, I will undertake to do so; and, first, I wish to compliment the essayist upon his careful preparation of the paper, and particularly upon the careful and systematic arrangement of the history, pathology, and symptomatology of the disease.

While the paper does not deal very fully with the problem of the treatment of the malady under consideration, yet it alludes to both medical and surgical interference. In illustration of the value of this side of the problem I wish to relate a statement made to me by a practitioner living some thirty miles east of Omaha while I was in attendance at a meeting of the Iowa and Nebraska Associations in Omaha last week. He said that the treatment of actinomycosis was a profitable phase of his business; that he had been called to treat some ten or eleven cases, in one bunch, on the day previous to the meeting, and that he received five dollars for each case, the price current in that section; that he had received several such calls recently, and you can imagine that that sort of business would make him feel in good humor. Hence, I feel that the practical side of this subject is of special importance to the practitioner in the country.

The value of undertaking treatment will depend upon one's point of view. If the animals can be rendered marketable the practitioner will hold that it is worth while, as also will his client. The meat-inspector will find that while the external lesions have been destroyed or removed the internal lesions are still present, and would hold that treatment was of no great value.

Relative to the employment of iodine internally administered, I wish to report some cases that we treated in the stockyards at Omaha and slaughtered to determine the results. In the cases where the soft parts alone were involved the actinomycotic growths disappeared, also the tumefactions produced by them, and where the bony tissues were invaded the size of the tumors was very greatly lessened, probably owing to the resorption of the fibrous tissue formations, which are always found surrounding such bony growths. In one case in which there was a characteristic antinomycotic growth in the maxillary sinus it was noted that while the new formation was not removed, yet it seemed to be shrivelled and changed in its appearance, giving the impression that the actinomyces had been destroyed.

Dr. Verschelden: I am very much interested in this question of the treatment of actinomycosis with iodide of potassium, and particularly am I anxious to know whether such cases will pass the inspector; whether the inspector can tell as to whether or not a case has been treated with iodide of potassium, and that in case he knows that it has been so treated, if he will pass it?

Dr. Stewart: In answer to Dr. Verschelden's question I would say that it very much depends on the inspector, and whether he be a Federal inspector or a municipal inspector, also somewhat upon his individual ideas.

Dr. Verschelden: The farmer in my vicinity always puts the question: Can they be so treated that they will pass the government inspection? If they can be cured so that they will pass the government inspection, then it will pay to have them treated, and I am particularly anxious to know what is done with these cases in Kansas City.

Dr. Milnes: It has fallen to my lot to pass upon a large number of these cases in the Kansas City yards, and as we are provided with suitable chutes for confining animals, the examination is quite critical. In cases where the lesions in soft parts have been properly and successfully treated, and only small thickenings remain to show that disease has some time been present, the cases are passed; but in other cases they are held for special post-mortem examination. Experience has shown, however, that in many cases where the external lesions are properly cured there are found, upon post-mortem examination, lesions of the internal organs. Farmers often follow these cases from the scales, where they have been detected by the government officials, to the special government retaining pen, to ascertain, and if possible influence, the final decision in their favor. In many cases the lesions are confined to the external soft parts, and it would seem proper and advisable for practitioners to undertake their treatment, and profitable to the farmer to have them treated, and if I were in practice I would advise their treatment.

Dr. Moore: In the treatment of these cases with iodide of potassium internally expense is quite a factor, the cases usually requiring a pound or more of the drug, which is quite expensive. So large a quantity need not be employed if during treatment the animal be not given foods containing large quantities of starch. Four ounces of the drug will suffice if the diet is restricted in the manner indicated.

Dr. Verschelden: I understood the essayist to hold that there was a great similarity between actinomycosis and fistula of the withers, and I wish to inquire if he ever treated fistula of the withers with iodide of potassium, and what sort of success he had?

Dr. Wright: My statement was not based upon experience, but entirely upon theory. I do not know that the organism producing fistula of the withers has been determined; it had seemed to me that the disease processes were quite similar as to formation of fistulæ, the character of the pus, slow healing, etc.

Dr. Verschelden: I was prompted to ask the question because of a case occurring in my practice. The case was suffering from what I concluded was crotalism, and was placed under the iodide treatment. As the animal also was afflicted with fistula of the withers, I noted that this disease seemed to be favorably influenced by the iodine.

Dr. Ernst: In the vicinity of Leavenworth, Fleming's lump-jaw cure has become quite popular with the farmers, as they seem to employ it quite successfully. I would like to know if the inspectors can tell whether cases have been treated with this remedy or not, and whether cases treated with this agent will pass the inspection.

Dr. Milnes: It is not possible to tell when the animals are marketed whether they have been treated by the Fleming lump-jaw remedy or some other. Many cases present the appearance of having been treated by some acid which leaves a large cicatrix, and when applied to the diseased bone there remains a large, ragged ulcer. When injected to the bottom of abscesses in the soft parts destruction of the secreting membrane takes place and perfect healing follows.

